# Biomarkers of fatigue related to adjuvant chemotherapy for breast cancer: evaluation of plasma and lymphocyte expression

**DOI:** 10.1186/s40169-015-0051-8

**Published:** 2015-02-14

**Authors:** Felipe M Cruz, Bruna A Munhoz, Beatriz CA Alves, Flavia S Gehrke, Fernando LA Fonseca, Renata K Kuniyoshi, Daniel Cubero, Luke J Peppone, Auro del Giglio

**Affiliations:** Discipline of Oncology and Hematology, ABC Foundation School of Medicine, Av. Príncipe de Gales, 821, Santo André, 09060-650 SP Brazil; Department of Surgery, University of Rochester, Rochester, 14642 NY USA

**Keywords:** Fatigue, Biomarkers, TGF-β, Breast Cancer, Chemotherapy, IL1-ra

## Abstract

**Background:**

Fatigue is common in cancer patients receiving adjuvant chemotherapy. To further understand the mechanism of fatigue and search for potential biomarkers, we conducted this prospective study. Methods

We enrolled breast cancer (BC) patients before their first adjuvant Adriamycin-based chemotherapy cycle. Patients responded to the brief fatigue inventory (BFI) and Chalder fatigue questionnaires and had their blood drawn for both plasma evaluation and evaluation of the peripheral mononuclear cell fraction (PMNCF) mRNA expression of various biomarkers. We evaluated FSH, LH, estradiol, DHEA, DHEAS, IL6, IL2, ILIRA, IL1β, CRP, Cortisol in the plasma and IL2, IL10, IL6, TGF-β, KLRC1, TNF, BTP, SNCA, SOD1, BLNK, PTGS2 and INF γ expression in the PMNCF.

**Results:**

11 patients did not exhibit an increase in their BFI scores and served as controls, whereas 32 patients exhibited an increase in their BFI scores compared with the baseline scores. From the biomarkers we evaluated in the PMNCF, the only one significantly associated with fatigue was TGF-β (p = 0.0343), while there was a trend towards significance with KLRC1 (p = 0.0627). We observed no evidence of significant associations of any plasma biomarkers with the development of fatigue. However when we analyzed patients with more severe fatigue, plasma IL1-RA levels correlated directly with higher fatigue scores (p = 0.0136).

**Conclusions:**

We conclude that fatigue induced by chemotherapy in BC patients is associated with changes in IL1-ra plasma levels and in TGF-β lymphocyte expression. Its mechanism may be different than that observed in long-term BC survivors or that induced by radiation therapy.

**Trial registration:**

NCT02041364 [ClinicalTrials.gov]

## Background

Breast cancer is the most frequently diagnosed cancer and the leading cause of cancer death among females, accounting for 23% of total cancer cases and 14% of cancer deaths [1]. The use of adjuvant systemic therapy is responsible, at least in part, for the reduction in cause-specific mortality from breast cancer [2]. The decision to use adjuvant chemotherapy for non-metastatic disease takes into account tumor histology, expression of estrogen and progesterone receptors, amplification of human epidermal growth factor receptor 2 (HER 2), tumor size, and nodal status [3].

The backbone of adjuvant chemotherapy treatment consists of a regimen which contains taxanes and anthracyclines and which is associated with the reduction in the risk of overall mortality when compared with a regimen not using these drugs [4]. However, chemotherapy carries some side effects that may worsen a patient’s quality of life.

Cancer-related fatigue (CRF) is defined as a distressing, persistent, subjective sense of physical, emotional, and/or cognitive tiredness or exhaustion related to cancer or cancer treatment that is not proportional to recent activity and differs from the normal fatigue that accompanies everyday life, which is usually temporary and relieved by rest [5,6]. CRF is a very common symptom in cancer patients at all stages of the disease’s evolution from its diagnosis and persists even to many years after the end of the disease [6,7]. A majority of patients will experience some level of fatigue during their course of treatment and approximately one-third will have persistent fatigue many years post-treatment [8,9]. However, before assuming that fatigue is related to prior treatment for breast cancer, treatable reasons for this symptom should be ruled out, including anemia, thyroid dysfunction, pain, depression and lack of sleep.

The mechanisms responsible for this condition are poorly understood. Major obstacles to defining the relevant pathophysiology of this symptom include the inherent subjectivity of fatigue, the difficulty in establishing objective behavioral correlates and the wide variety of conditions unrelated to cancer or its treatment that contribute to fatigue [10]. Some pathophysiologic hypotheses have been proposed for CRF causes such as disrupted circadian rhythms [11], loss of muscle mass [12], chronic stress response mediated through the hypothalamic pituitary axis [12,13], systemic inflammatory response [14-17] and pro-inflammatory cytokines [18,19]. However, none of these hypotheses have been proved yet.

The role of inflammatory cytokines may be based upon several lines of evidence. Non-oncologic patients with Chronic Fatigue Syndrome have increased levels of pro-inflammatory cytokines such as IL1 beta, IL 1 receptor antagonist and TNF-alpha [20,21], and fatigue is a major side effect of cancer patients receiving interleukins, TNF-alpha and interferon [22-24].

Patients with fatigue also present inflammatory changes manifested by increased levels of several cytokines (IL1, IL6) [20,25,26] and other inflammatory markers such as the C-reactive protein (CRP) [27]. Indeed, tumor cells are located in both the primary tumor and metastases in direct proximity to stromal cells such as lymphocytes and macrophages which secrete various cytokines. In turn, the proliferation of nearby tumor cells ADDIN BEC{Seruga et al., [28], Nat Rev Cancer, 8, 887–99} may be positively stimulated by this autocrine loop. These cytokines may also contribute to the production of some of the more common symptoms of widespread systemic cancer, for example, fatigue and wasting [28]. Lanmark and colleagues described alterations in the signaling pathways of B lymphocytes in patients with breast cancer with and without fatigue [29]. More recently, cancer-related fatigue (CRF) was associated to the over-expression of alpha-synuclein (SNCA), which is present in diseases such as Parkinson’s [30] and is involved in some cases of hereditary amyloidosis [31]. This may indicate the potential role of inflammatory pathways in the development of CRF [32].

Since patients undergoing adjuvant chemotherapy for breast cancer often manifest fatigue [33], this scenario can be considered as providing a true experimental model for research of biomarkers of this symptom. The identification of potential fatigue biomarkers may be helpful to establish predictive markers of response to therapy in chemotherapy induced fatigue trials. This work explores the possibility of evaluating changes in cytokine production observed both in the plasma and mononuclear fraction of the peripheral blood of patients with breast cancer and fatigue in order to ascertain if there are specific patterns of cytokine expression that may be identified in these patients.

## Methods

### Clinical study

Patients with stage I, II and III breast cancer undergoing adjuvant systemic chemotherapy based on the use of anthracyclines were screened before the start of their treatment. We excluded patients with any condition which may cause fatigue such as hypothyroidism, personal history of self reported depression, anemia, decompensated heart failure or hypertension (systolic pressure ≥ 140 mmHg and or diastolic pressure ≥ 90). Our Institutional Ethics Committee approved this study.

Patients who agreed to participate by signing an informed consent form received their first cycle of chemotherapy. Patients whose level of fatigue as measured by the Brief Fatigue Inventory (BFI) increased by at least one point after having received the first cycle of chemotherapy were considered as having manifested fatigue induced by chemotherapy.

The patients whose fatigue did not worsen following the first cycle of chemotherapy served as controls.

The patients completed questionnaires to evaluate their fatigue (BFI [34] and Chalder [35]) and had blood drawn before the start of chemotherapy and before the second cycle of chemotherapy. The two questionnaires had previously been validated for use in Portuguese [36,37].

We chose, based on a literature review, the following biomarkers to be evaluated in the serum as well as their expression in peripheral blood mononuclear cells: IL-2, IL-10, SOD1, BLNK, PTG S2, KLRC1, TGB1, IL 6, IFNG, TNF, SNCA, BTP.

### Analysis of gene expression

#### RNA extraction

We reviewed the relevant literature to select potential plasma biomarkers and genes whose expression in lymphocytes could relate to the process of fatigue produced by chemotherapy. Blood samples were collected Upon venous puncture. Tubes containing EDTA for RNA extraction were collected. The total RNA of the patients included in this study was extracted from the mononuclear fraction of peripheral blood (MFPB) using the QIAamp RNA Blood Mini Kit (Qiagen, cat. no. 52304). The mass and purity of RNA were determined by spectrophotometry at wavelength from 260 to 280 nm. Samples with a 260/280 ratio between 1.8 and 2.0 were considered valid. To exclude trace gDNA, the RNA samples were treated with DNase I Amplification Grade (Invitrogen, catalog No. 18068–015), according to the manufacturer’s protocol. The total RNA of the samples was stored at −80°C until conversion into cDNA.

#### CDNA synthesis

CDNA was synthesized from 500 ng of total RNA using the SuperScript III First-Strand Synthesis SuperMix for qRT-PCR kit (Invitrogen, Cat. no. 11752–250).

#### Amplification reaction

The Δ amplification reactions using hydrolysis probes present in the customized plates were performed in a 7500 thermocycler (Applied Biosystems) in a final volume of 20 μL containing 1X TaqMan Master Mix (Applied Biosystems, PN4391016) and 2.0 μL of cDNA diluted 10X. The thermal cycling conditions used were an initial step of 2 min at 50°C and 10 min at 95°C followed by 40 cycles of 15 sec at 95°C and 1 min at 60°C. Gene expression was evaluated using the standard curve method.

Amplification reactions of 12 differentially expressed genes identified between the constants on the plates with a hydrolysis probe were performed for 45 patients using the SYBR Green system (QuantiFast SYBR Green PCR Kit, Qiagen, Cat no. 204057). The amplification reactions occurred in a final volume of 15 μL containing 1X SYBR Green buffer, 0.25 μM primers (for IL2, IL10, SOD1, BLNK and PTGS2 genes) or 0.20 μM primer (for KLRC1, TGB1, IL6, IFNG, TNF, SNCA and BTP genes) and 2.0 μL of 10X diluted cDNA. The thermal profile used was an initial step of 95°C for 10 minutes and 40 cycles of 95°C for 15 seconds and 60°C for 60 seconds (for IL2, IL10, SOD1, BLNK and PTGS2 markers) and an initial step of 95°C for 10 minutes and 40 cycles of 95°C for 15 seconds and 61°C for 60 seconds for the remaining markers.

Specific primers for validation: the specific primers for each target gene in this study were designed using the Primer-Blast tool, available at http://www.ncbi.nlm.nih.gov/tools/primer-blast/index.cgi?LINK_LOC=BlastHome (Table 1).

**Table 1:** **Characteristics of the specific primers used to validate the gene expression results**

**Gene**	**Sequence (5’ - 3’)**	**Amplicon (bp)**	**No. access**
IL2	F-CCCAAGAAGGCCACAGAACT	125	NM_000586.3
	R-TTGCTGATTAAGTCCCTGGGT		
IL10	F-GCTGAGAACCAAGACCCAGA	141	NM_000572.2
	R-ATTCTTCACCTGCTCCACGG		
SOD1	F- GGTGGGCCAAAGGATGAAGA	129	NM_000454.4
	R- GCCAATGATGCAATGGTCTCC		
BLNK	F- AACAGGAAGCTGGCGTTCTC	124	NM_013314.3
	R- TGGCCAGAGCTTTTCCGAAT		
PTGS2	F- TGAGTGTGGGATTTGACCAGT	128	NM_000963.2
	R- GTGCACTGTGTTTGGAGTGG		
KLRC1	F- ACCATCCTCATGGATTGGTGT	163	NM_002259.4
	R- TGAAGATCCACACTGGGCTG		
TGB1	F-CTGACTGCTCTGGCTTCCTC	176	NM_002704.3
	R- TGGGTTCCTTTCCCGATCAC		
IL6	F-CCAGAGCTGTGCAGATGAGT	174	NM_000600.3
	R- AGCTGCGCAGAATGAGATGA		
IFNG	F- TGGAAAGAGGAGAGTGACAGA	122	NM_000619.2
	R- TCTTCCTTGATGGTCTCCACAC		
TNF	F- AGAGGGAAGAGTTCCCCAGG	123	NM_000594.3
	R- CCTCAGCTTGAGGGTTTGCT		
SNCA	F- ATGTTGGAGGAGCAGTGGTG	134	NM_000345.3
	R- CTGTGGGGCTCCTTCTTCAT		
BTP	F- CAGCACCTACTCCGTGTCAG	142	NM_000954.5
	R-CTTTAACTCAGCCCTGGGGG		

QPCR results analysis: The results of gene expression obtained with hydrolysis probe plates were analyzed using the standard curve method, and the gene expression results in the validation phase were obtained using the formula 2^-ΔCq^.

#### Plasma dosages

Blood samples were collected by peripheral venipuncture. Approximately 12.0 mL of whole blood was collected from each patient at the times proposed in the clinical protocol. After collection, the samples were centrifuged, and the plasma was obtained for determination of hormones and interleukins. The FSH, LH, estradiol, DHEA, DHEAS and cortisol hormones were determined by chemiluminescent enzyme immunometric assay using an Immulite 1000 luminometer. The IL6, IL2 and usCRP cytokines were determined by competitive enzyme assay and also from a chemiluminescent reading using an Immulite 1000 luminometer. The remaining components, such as ILIRA and IL1β, were determined by enzyme immunoassay and an optical density reading using a Labotech ELISA semi-automated reader. All the determinations were evaluated in duplicate following best practice in clinical analysis.

#### Statistical analysis

We evaluated comparisions between continuous variables and categorical variables using the Kruskal-Wallis test when a continuous variable distribution was not normal and we employed the ANOVA test if its distributions was normal. The normality of the distribution of continuous variables was evaluated using the Kolmogorov-Smirnov test. P values of less than 0.05 were considered significant. The Prism® program version 6d (GraphPad Software, La Jolla California USA, www.graphpad.com) was used to perform all the statistical calculations in this study.

## Results

A total of 43 female patients were included. The BFI questionnaire fatigue score of 11 of these patients did not increase, and they were therefore considered as controls. Patients whose BFI score increased at least 1 point after the first cycle of chemotherapy constituted the fatigue group (n = 32).

We observed a statistically significant difference between the BFI global scores of the fatigue group and those of the control group(Mean BFI Fatigue = 44,2 range: 4–89 vs. Mean BFI control = 0,9, range 0–2, p = 0,0001). The classifications of the severity of fatigue by Chalder and BFI were similar. The clinical characteristics of the patients are shown in Table 2.

**Table 2:** **Clinical characteristics of recruited patients**

	**Patients with fatigue (randomized)**	**Patients without fatigue (control)**	**p**
Age (mean ± standard deviation)	48.9 ± 9.6	55.7 ± 3.8	0,08
Race			
African-American	26(81.3%)	9 (81.8%)	0.8
Caucasian	2 (6.2%)	2 (18.2%)	0.8
Stage			
I	5 (15.6%)	3 (27.3%)	0.33
II	15 (46.9%)	3 (27.3%)	0.10
III	12 (37.5%)	6 (45.4%)	0.40
Body Mass Index	23.1 ± 2	22.3 ± 1,2	p = 0.8
Histological Type			
Ductal Carcinoma	28 (87.4%)	11 (100%)	0.6
Lobular Carcinoma	2 (6.3%)	-	-
Metaplastic Carcinoma	2 (6.3%)	-	-
Hormone Receptors/HER2			
Hormone Receptor - positive, HER2 -positive	9 (28.1%)	3 (27.3%)	0.99
Hormone Receptor - positive, HER2 - negative	13 (40.7%)	5 (45.4%)	0.80
Hormone Receptor -negative, HER2 positive	1 (3.1%)	1 (9.1%)	1
Triple-negative	9 (28.1%)	2 (18.2%)	0.70
Type of Chemotherapy			
Adjuvant	18 (56.2%)	5 (45.5%)	0.40
Neoadjuvant	14 (43.8%)	6 (54.5%)	0.70
Comorbidities			
Lack of Comorbidities	26 (81.3%)	9 (81.8%)	0.80
Arterial hypertension	2 (6.2%)	2 (18.2%)	0.80
Diabetes mellitus	4 (12.5%)	0	-
Menopausal Status			
Premenopausal	13 (40.6%)	9 (81.8%)	0.10
Postmenopausal	19 (59.4%)	2 (18.2%)	0.20

### Evaluation of plasma level and biomarker lymphocyte expression in patients before and after chemotherapy who developed or did not develop fatigue

**Figure 1:**
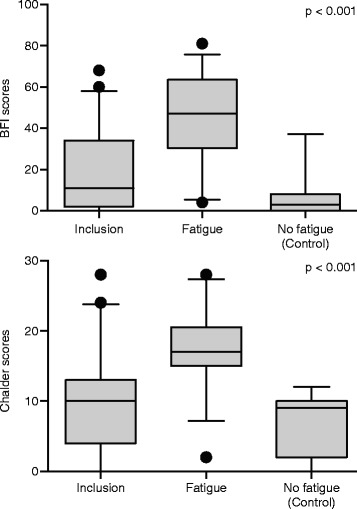
**The significant variations in fatigue in the 3 groups evaluated in Figures** **2** **and** **3** **, namely patients at the screening stage (inclusion, all patients), including all 43 patients (32 patients whose fatigue worsened after chemotherapy and 11 patients whose fatigue did not worsen after chemotherapy and who served as controls for this stage of data analysis).** The black dots represent the outliers.

**Figure 2:**
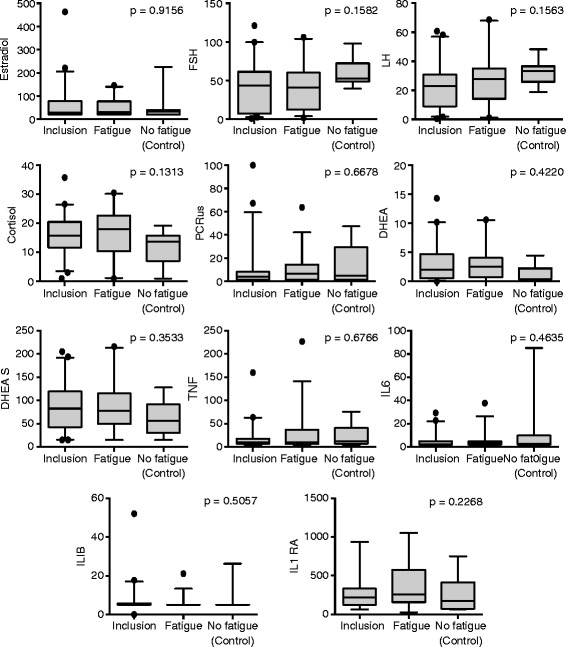
**Evaluation of plasma analytes in patients in the inclusion phase whose fatigue worsened and in patients whose fatigue did not worsen after the first cycle of chemotherapy (controls).** The black dots represent the outliers.

**Figure 3:**
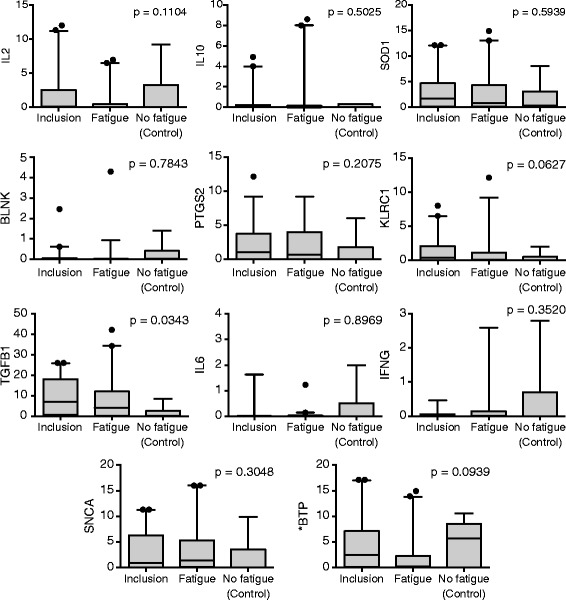
**Evaluation of lymphocyte expression of selected genes in the first phase of the study in patients in the inclusion phase whose fatigue deteriorated and in patients whose fatigue did not worsen after the first cycle of chemotherapy (controls).** The black dots represent the outliers.

Figure 1 reveals the significant variations of fatigue in each group being evaluated in Figures 2 and 3, which in turn illustrate the changes observed in the biomarkers evaluated in the blood and lymphocytes, respectively, for all the study patients.

No variation in any of the analytes evaluated was observed in the plasma (Figure 2). We did however observe a significant variation in the lymphocyte expression of TGFB1 in these groups and a trend toward significance for the expression of KLRC1 (p = 0.06) and BTP (p = 0.09).

**Figure 4:**
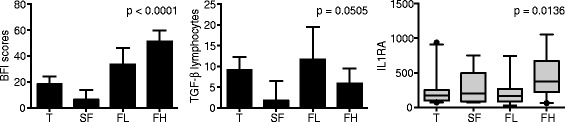
**Patients who developed fatigue where separated in two groups: those who had higher BFI fatigue scores (HF), ie who had an increase equal or above 1 standard deviation (20 points) in their BFI scores as compared to the triage (T) level and 2) those who had lower increases in their BFI scores (FL).** Also shown are those patients who had a decrease in their BFI scores after the first cycle of chemotherapy who were considered as controls (C).

As an exploratory analysis, we stratified patients by the increase in their BFI scores in two groups: 1) who had more severe fatigue (ie had at least one Standard deviation (SD) increase in their BFI scores after the first cycle of chemotherapy) and 2) those who had lower increases in their BFI scores. We observed then a positive association with ILI-ra (p = 0.0136) and with TGF-β (p = 0.0505). Interestingly TGF-β levels were higher in less severe fatigued patients (Figure 4).

## Discussion

We observed no plasma biomarker in our study that was statistically significantly associated with the presence of chemotherapy-induced fatigue. However, in an exploratory analysis of patients who were more fatigued, we identified plasma IL1-ra as a potential biomarker of chemotherapy induced fatigue. Interestingly, when evaluating the lymphocyte expression of several potential markers, we observed a significant correlation between fatigue and expression levels of TGFB-1 and a trend for KLRC1 and BTP.

The TGFB-1 gene, also known as PPBP, encodes a protein that is a platelet derived growth factor and exhibits intense chemotactic activity for neutrophils and stimulates DNA, glycolysis, mitosis, cAMP intracellular accumulation and prostaglandin E2 synthesis, among other activities. Among our patients, we observed that those who developed fatigue after the first cycle of chemotherapy exhibited higher levels of this gene than the patients who did not develop fatigue. Kennedy and colleagues also observed high levels of TGFB-1 in the peripheral blood of patients with chronic fatigue which was associated with an increased rate of neutrophil apoptosis when compared with controls without chronic fatigue [38]. Interestingly in our exploratory analysis, unlike IL1-ra, TGFB-1 seems to be associated with less severe degrees of fatigue.

The KLRC1 gene encodes a protein expressed in NK cells and, in association with KLRD1/CD94, this protein is involved in the recognition of HLA class I molecules by NK cells. NK cells play an important role in the immune system and in the immune response to tumors. Aspler [39], who studied gene expression of peripheral blood cells of patients with chronic fatigue, also described a negative association between chronic fatigue and the presence of genes expressed in NK cells. In our study, patients who did not exhibit fatigue after the first cycle tended to express less KLRC1 than at the screening stage.

The BTP gene encodes a protein known as beta-trace protein (BTP) or PTGDS that is homologous to the glutathione-independent prostaglandin synthetase enzyme that catalyzes the conversion of prostaglandin H2 to prostaglandin D2 (PGD2). PGD2 acts as a neuromodulator and a trophic factor in the central nervous system. In addition to these functions, PGD2 is also important for smooth muscle contraction and is an important inhibitor of BTP platelet aggregation (PTGDS) that may also be involved in the regulation of non-REM sleep [40]. In our study, we observed that patients without fatigue after the first cycle of chemotherapy had a tendency to exhibit increased levels of BTP (PTGDS), suggesting that higher levels of this substance could be associated with protection from fatigue.

The significant positive association between higher fatigue scores and IL-1ra that we found in an exploratory analysis has also been reported by others [17,18,41,42]. This association, however, appears to be counterintuitive [32]. Since IL-1ra inhibits IL-1β and other members of the IL-1 family by binding to their target cell receptors without agonist activity it would be expected to have an anti-inflammatory action. Whether the increase in IL1-ra that we observed was a reaction to even more increased IL1-β levels (which we were unable to document in this study) or to high levels of other IL-1 family members is unknown [32].

While this study was conducted in a prospective manner with multiple biomarkers, several other issues should be considered in interpreting the results. Primarily, the results are not generalizable to all cancer patients with fatigue. In addition, a limitation of our study is that it consisted of only a small sample of breast cancer patients receiving Adriamycin-based chemotherapy. It is possible that other chemotherapy regimens or cancer of other sites could have different biomarker profiles than that which we observed. The number of controls who did not experience fatigue was also limited. Because fatigue is extremely prevalent in cancer patients receiving chemotherapy, controls who did not have an increase in fatigue scores after the first cycle of chemotherapy were few. This small number of controls consisting of only 11 patients may have underpowered our study to detect potential smaller, albeit significant biomarker differences, between fatigued and non-fatigued patients.

The aim of our study was to evaluate potential biomarkers of chemotherapy induced fatigue. That is the reason we used cancer patients without an increase in their fatigue global scores after having received chemotherapy as controls. We divided our patients in two groups, those who developed fatigue and those who did not (the controls) after the first cycle of chemotherapy, in order to be able to conduct an exploratory analysis to identify potential biomarkers of fatigue. In order to exclude spurious findings due to the inclusion in the fatigue group of patients with very modest increases in their BFI scores, we also evaluated separately the group of patients who had the largest increases in their BFI scores and compared them with the controls. In fact, the evaluation of these more severely fatigued patients allowed us to identify biomarkers which will need to be validated in the future in a larger sample of patients.

Fatigue linked to chemotherapy appears to play a minor role in the hypothalamic pituitary axis and even in several inflammatory cytokines such as IL-6, IL-1, IL2 as described in other studies [32,43,44]. It is possible that its physiopathology is different from fatigue induced by radiotherapy [45] or that observed in survivors of cancer months or years after treatment [17,29]. We believe that further studies, focused on this patient population and with a broader panel of biomarkers, may be capable of unraveling possible mechanisms for the genesis of this symptom and validate our findings.

## Conclusions

We conclude that fatigue induced by chemotherapy in BC patients is associated with changes in IL1-ra plasma levels and in TGF-β lymphocyte expression. Its mechanism may be different than that observed in long-term BC survivors or that induced by radiation therapy.
